# Apigenin inhibits pancreatic cancer cell proliferation through G2/M cell cycle arrest

**DOI:** 10.1186/1476-4598-5-76

**Published:** 2006-12-29

**Authors:** Michael B Ujiki, Xian-Zhong Ding, M Reza Salabat, David J Bentrem, Laleh Golkar, Ben Milam, Mark S Talamonti, Richard H Bell, Takeshi Iwamura, Thomas E Adrian

**Affiliations:** 1 Department of Surgery and Robert H. Lurie Comprehensive Cancer Center, Feinberg School of Medicine, Northwestern University, Chicago, Illinois, USA; 2 Department of Surgery, Miyazaki University School of Medicine, Miyazaki, Japan; 3 Department of Physiology, United Arab Emirates University, Al Ain, UAE

## Abstract

**Background:**

Many chemotherapeutic agents have been used to treat pancreatic cancer without success. Apigenin, a naturally occurring flavonoid, has been shown to inhibit growth in some cancer cell lines but has not been studied in pancreatic cancer. We hypothesized that apigenin would inhibit pancreatic cancer cell growth *in vitro*.

**Results:**

Apigenin caused both time- and concentration-dependent inhibition of DNA synthesis and cell proliferation in four pancreatic cancer cell lines. Apigenin induced G2/M phase cell cycle arrest. Apigenin reduced levels of cyclin A, cyclin B, phosphorylated forms of cdc2 and cdc25, which are all proteins required for G2/M transition.

**Conclusion:**

Apigenin inhibits growth of pancreatic cancer cells through suppression of cyclin B-associated cdc2 activity and G2/M arrest, and may be a valuable drug for the treatment or prevention of pancreatic cancer.

## Background

Despite attempts of resection and adjuvant therapy, patients diagnosed with pancreatic cancer continue to have a poor prognosis. Many chemotherapeutic agents have been used to treat pancreatic cancer without particular success. Novel treatments to inhibit cancer cell proliferation with less toxicity are needed. Flavonoids are a class of polyphenolic compounds which display a variety of biologic activities. There has been recent interest in using flavonoid derivatives therapeutically as anticancer drugs. At pharmacological levels, various naturally occurring flavonoids have been shown to be cancer-protective in a variety of animal models. Flavonoid derivatives, such as flavopiridol and genistein, have been shown to be safe in human trials[1,2] and are being assessed as chemotherapy drugs in phase II clinical trials for advanced solid tumors [3,4].

Flavonoids have shown antitumor activity for a number of cancer cell types [5,6]. This is mediated by different types of cell cycle arrest and the induction of apoptosis in several tumor cell lines [7-12]. Genistein, a naturally occurring isoflavone, has shown antitumor activity in pancreatic cancer models [13]. Apigenin, an isoconformer of genistein, has shown more potent growth inhibition in several cancer cell lines [6]. Apigenin has been shown to possess anti-inflammatory effects, free radical scavenging properties, and anti-carcinogenic effects [14]. It has been shown to possess growth inhibitory properties in several cancer lines, including breast, colon, skin, thyroid, and leukemia cells [15-19], but has never been studied in pancreatic cancer. We hypothesized that apigenin would inhibit pancreatic cancer cell growth *in vitro*, and further aimed to delineate the mechanism involved.

## Results

### Apigenin inhibited DNA synthesis

**Figure 1:**
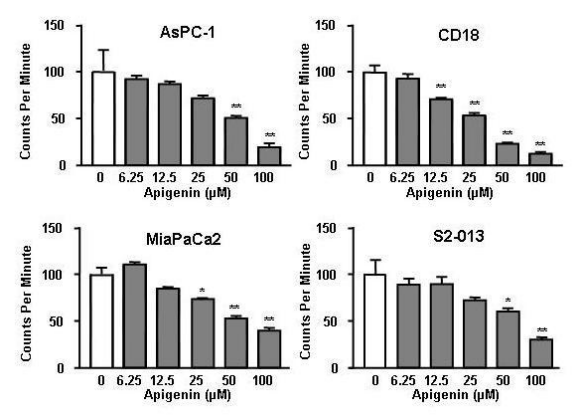
**Effects of apigenin on DNA synthesis in human pancreatic cancer cells**. Apigenin caused significant concentration-dependent inhibition of DNA synthesis in all four cell lines studied: AsPC-1 [*F *(5,42) = 10.12, P < 0.0001; P = 0.01 with 50–100 μM]; CD18 [*F *(5,54) = 38.47, P < 0.0001; P < 0.01 with 12.5–100 μM]; MIA PaCa2 [*F *(5,72) = 8.698, P < 0.0001; P < 0.05 with 25 μM and P < 0.01 with 50–100 μM]; S2-013 [*F *(5,72) = 4.099, P < 0.0025; P < 0.05 with 50 μM and P < 0.01 with 100 μM]. Data represent results from at least three separate experiments each performed in triplicate, expressed as a percentage of control.

Apigenin caused a concentration-dependent (6.25–100 μM) inhibition of thymidine incorporation in all four cell lines studied (Figure 1). At 24 hours, 100 μM apigenin caused an approximately 50% inhibition of thymidine incorporation in each cell line. The inhibition of proliferation was statistically significant (P < 0.01) at 100 μM apigenin in all cell lines.

### Apigenin inhibited cell proliferation

**Figure 2:**
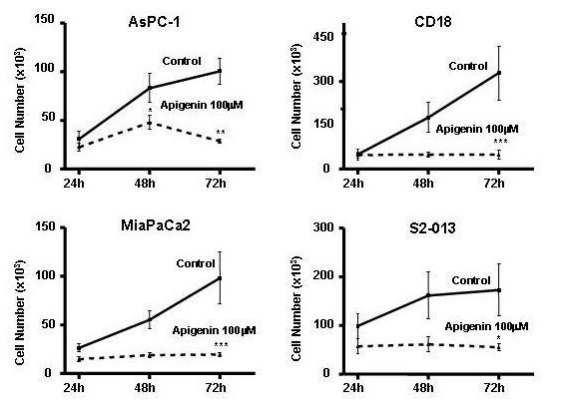
**Time-course effects of apigenin on cell number of human pancreatic cells**. Apigenin significantly inhibited the increase in cell number of each cell line over time: AsPC-1, [*F *(5,59) = 10.65, P < 0.0001], P < 0.05 at 48 h and P < 0.001 at 72 h; CD18, [*F *(5,38) = 5.641, P < 0.0006], P < 0.001 at 72 h; MIA PaCa2, [*F *(5,65) = 6.127, P < 0.0001], P < 0.001 at 72 h; S2-013, [*F *(5,60) = 2.589, P < 0.0347], P < 0.05 at 72 h. Data represent results from at least three separate experiments each performed in triplicate, expressed as cell number/well. *= P < 0.05; **= P < 0.01; ***= P < 0.001 compared with control.

The inhibition of DNA synthesis correlated with a decrease in cell number over a 72-hour period when compared with control. The cell number in untreated cells increased every 24 hours in almost every cell line while cell number of apigenin-treated cells increased at a slower rate, or decreased (Figure 2). The differences in cell number were statistically significant (P < 0.05) at 72 hours in all four cell lines. During the first 24 hours, no significant difference was seen between cells treated with apigenin and controls. Only viable cells were counted so that cytotoxicity did not play a role.

### Apigenin induced G2/M phase cell cycle arrest

**Figure 3:**
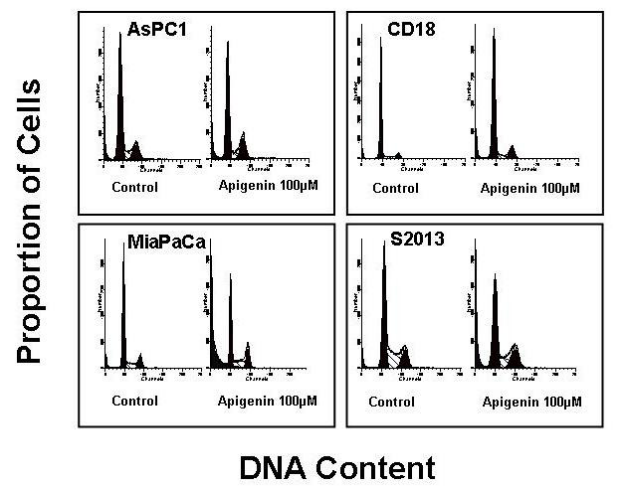
**Effects of apigenin on the proportion of cells in different phases of the cell cycle**. Apigenin caused G2/M arrest in all four human pancreatic cancer cell lines studied. Similar results were seen in four different experiments.

**Figure 4:**
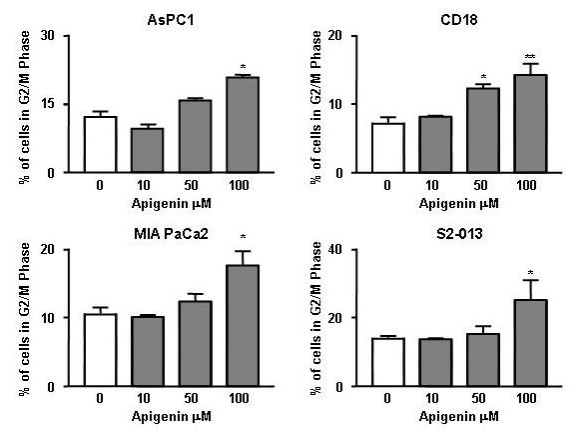
**Concentration-dependent effects of apigenin on the cell cycle**. Higher concentrations of apigenin cause a concomitant increase in the percentage of cells in the G2/M phase. AsPC-1 [*F *(3,8) = 12.79, P < 0.002 (P < 0.01 with 100 μM)]; CD18 [*F *(3,8) = 9.504, P < 0.0128 (P < 0.05 with 50 μM and P < 0.01 with 100 μM)]; MIA PaCa2 [*F *(3,10) = 6.062, P < 0.0128(P < 0.01 with 100 μM)]; S2-013 [*F *(3,10) = 4.071, P < 0.0394 (P < 0.05 with 100 μM)]. *= P < 0.05; **= P < 0.01 compared with control.

In order to better understand the mechanism of inhibition of cell proliferation, the distribution of cells in the different phases of the cell cycle was analyzed following treatment with 100 μM apigenin for 24 hours. There were marked and consistent changes in the cell cycle at 24 hours. The number of cells in the G2/M phase increased in all four cell lines with a concomitant decrease in treated cells in the G0/G1 and S phase. The difference between the mean percentage of control and treated cells in the G2/M phase of four separate experiments was statistically significant in all four cell lines (AsPC1: P < 0.01, CD18: P < 0.009, MiaPaCa2: P < 0.04; S2013: P < 0.03). Figure 3 shows the results of one experiment representative of all cell cycle experiments. The concentration-dependent effects of apigenin on the proportion of cells in the G2/M phase of the cell cycle are shown in Figure 4.

### Apigenin changed expression of proteins involved in the G2/M phase transition

**Figure 5:**
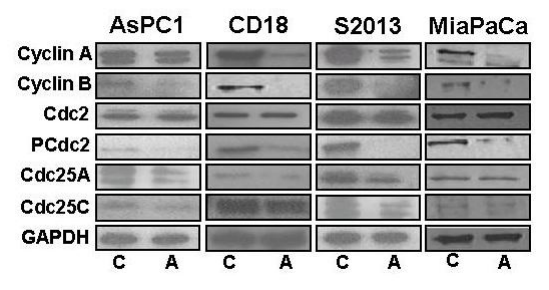
**Effects of apigenin on proteins involved in the G2/M cell cycle phase transition**. Expression of both cyclin A and cyclin B were both decreased in apigenin-treated cells compared to controls. While the expression of the cdc2 protein was unchanged, levels of phosphorylated cdc2 were decreased in treated cells. Expression of cdc25A and cdc25C were also decreased in treated cells compared to controls.

All four pancreatic cancer cell lines were treated with 100 μM apigenin for 24 hours and expression and phosphorylation of proteins involved in the G2/M transition were observed through western blotting (Figure 5). Expression of cyclin A and B was decreased in apigenin treated cells when compared to controls. While expression of cdc2 was unchanged, the levels of phosphorylated cdc2 were decreased in apigenin treated cells. Expression of cdc25A and cdc25C were also decreased in apigenin treated cells compared to controls.

## Discussion

Several mechanisms have been proposed for apigenin's effect on cell growth including modulation of the MAP kinase pathway [20,21], inhibition of ornithine decarboxylase [22], improvement in cell-cell communication through increased gap junctions [23], and cell cycle arrest. Cell-cycle checkpoints at G2/M as well as G1/S are critical in maintaining DNA integrity and regulating the passage of cells through the cell cycle. It is well known that loss of these checkpoints is involved in the transformation into and progression of cancer cells. A protein kinase complex consisting of a catalytic subunit, cdc2, and the cyclin B protein performs the central and rate-limiting function in the transition from G2 to M phase [24]. The cdc2/cyclin B complex responds to DNA damage and causes a delay in cell cycle progression to allow DNA repair before cells enter mitosis. The complex accomplishes this through phosphorylation of cytoskeleton proteins such as lamins and histone H1 [25]. Cdc2 binding to cyclin B in and of itself is not enough, however, for checkpoint progression. Dephosphorylation of cdc2 at the Tyr-15 site through cdc25c phosphatase is required for activation of the complex [24,26]. Cdc25A also contributes to the cellular phosphatase pool required to dephosphorylate cdc2 fully [27]. Cyclin A, known mainly for its role in G1/S transition, is also required for the entry of cells into mitosis [28,29].

In the current study, treatment of human pancreatic cancer cells with apigenin resulted in inhibition of DNA synthesis and cell proliferation through G2/M cell cycle arrest. A decrease in levels of cyclin A, cyclin B, cdc25 A and cdc25C all appear to be involved. It is not clear why levels of phosphorylated cdc2 were decreased given that reduced levels of cdc25c should have the opposite effect, however, the reduced levels of cyclin B appear to be the main mechanism involved in the arrest of cells at G2/M.

Our results are congruent with previous studies in which cell cycle arrest has been shown to result from apigenin treatment in other cell lines. G2/M arrest through inhibition of cyclin B associated cdc2 has been shown to occur in epidermal and fibroblast cells [8,30], breast cancer cells [31], oral cancer cells [32], melanoma [33], mouse keratinocytes [34], endothelial cells [35], prostate cancer cells [36], and colon cancer cell lines [16]. Interestingly, inhibition of cyclin B appears to be concentration-related as lower doses have less effect while higher doses (>70 μM) as we used in our study decrease cyclin B levels [34].

A considerable amount of attention has been given to the role of p53 in cell cycle checkpoint control. P53 blocks cells at G2/M through direct inhibition of cdc2 kinase [37]. All four cell lines used in this study contain mutated p53, which implies that the G2/M arrest seen after apigenin treatment is through a p53-independent pathway. Other studies have found this to be the case as well. Apigenin was found to inhibit growth in both p53 wild-type and mutated breast cancer cells [15], p53 mutated oral squamous cell cancer cells [32], and mutated colon cancer cell lines [16].

## Conclusion

To our knowledge there is no published data concerning apigenin in pancreatic cancer. Our results demonstrate that apigenin inhibits human pancreatic cancer cell growth *in vitro *through G2/M phase cell cycle arrest associated with decreased cyclin B-cdc2 activity. Taken together with previously published studies, apigenin appears to exert its inhibitory effects on cell cycle progression in other cell lines as well. Cancer cells with abnormal cell-cycle machinery may benefit from extrinsic cell-cycle regulators such as apigenin.

## Methods

### Materials

DMEM, trypsin-EDTA solution, and propidium iodide were purchased from Sigma (St. Louis, MO). Fetal bovine serum was purchased from Atlanta Biologicals (Norcross, GA). Apigenin was purchased from Calbiochem (Darmstadt, Germany) and dissolved in DMSO purchased from Sigma Chemicals (St. Louis, MO). Methyl ^3^H-Thymidine was purchased from Amersham (Arlington Heights, IL). The monoclonal mouse cyclin A, B, cdc25A, and GAPDH antibodies, and the polyclonal rabbit cdc25C antibody were purchased from Santa Cruz Biotechnology (Santa Cruz, CA). The monoclonal rabbit cdc2 and phospho-cdc2 (Tyr^15^) antibodies were purchased from Cell Signaling Technology, Inc. (Beverly, MA).

### Human pancreatic cancer cell lines and cell culture

We selected four human pancreatic cancer cell lines: AsPC-1, CD18, MIA PaCa2, and S2-013. These cell lines were purchased from American Type Culture Collection (Manassas, VA) with the exception of the S2-013 cell line, which was a generous gift from Dr. Takeshi Iwamura (Japan). Each cell line was grown in DMEM and plated as monolayers in medium supplemented with 10% fetal bovine serum in a humidified atmosphere of 95% O_2 _and 5% CO_2 _at 37°C. The cells were regularly seeded into 75-cm^2 ^flasks with media changes every second or third day. For experiments, cells were grown to 70% confluence, digested with trypsin-EDTA, and plated in 6-, 24-, or 48-well plates.

### DNA synthesis by methyl-^3^H thymidine incorporation

Cells were plated in 24-well plates at a concentration of 50,000 cells/well. After reaching 50% confluence, they were incubated in serum-free medium for 24 hours, which was then replaced with fresh serum-free medium with or without the appropriate treatments. After the required period of culture, cellular DNA synthesis was assayed by adding 0.5μCi methyl ^3^H-thymidine/well and incubating cells for another 6 hours. The cells were then washed twice with PBS, fixed with 10% trichloroacetic acid, and solubilized by adding 250 μl of 0.4 M NaOH to each well. Radioactivity, indicating incorporation of methyl ^3^H-thymidine into DNA, was measured by adding scintillation cocktail and counting on a scintillation counter (LKB RackBeta; Wallac, Turku, Finland).

### Cell proliferation assay

Cells were regularly seeded into three 6-well plates and incubated at 37°C for 24 hours. Cells were then cultured in serum-free medium for another 24 hours and treated in fresh serum-free medium with or without apigenin for 24, 48, and 72 hours. At the end of each time period, the cells were trypsinized to produce a single cell suspension, and the viable cell number in each well was counted using Guava Technologies' ViaCount Assay (Guava Technologies Inc, Hayward, CA).

### Analysis of cellular DNA content by flow cytometry

Cells were grown to 50% confluence in T75-cm^2 ^flasks, serum starved for 24 hours, and then treated with apigenin. At the end of the treatment, the cells were harvested with trypsin-EDTA solution to produce a single-cell suspension. Cells were then pelleted by centrifugation and washed twice with PBS. The cell pellets were then suspended in 0.5 mL PBS and fixed in ice-cold 70% ethanol at 4°C. The fixed cells were then centrifuged at ×300 g for 10 minutes and pellets washed with PBS. After resuspension in 1 ml PBS, the cells were incubated with 10 μL Rnase I (10 mg/ml) and 100 μL propidium iodide (400 g/mL) and shaken for 1 hour at 37°C in the dark. The samples were then analyzed by flow cytometry.

### Western blotting

Cells were seeded in T25-cm^2 ^flasks and grown to 60% confluence for 24 hours. The cells were serum starved for 24 hours. The cells were then placed in serum-free medium with or without 100 μM apigenin for 24 hours. The cells were lysed and protein concentrations determined with BSA as standard. Equal amounts of cell protein lysate were separated on 15% SDS-polyacrylamide gel electrophoresis (PAGE) and the protein transferred onto nitrocellulose membranes. After blocking with dried milk, membranes were incubated with the appropriate dilution of primary antibody. Membranes were then incubated with a horseradish peroxidase-conjugated secondary antibody. Proteins were detected using the enhanced chemiluminescence detection system.

### Statistical analysis

Data were analyzed by ANOVA with Dunnett's or Bonferoni's corrections for multiple comparisons, as appropriate. This analysis was performed with the Prism software package (GraphPad, San Diego, CA). Data were expressed as mean ± SEM.

## Authors' contributions

MU carried out the cell culture work, thymidine incorporation, cell proliferation assays, flow cytometry, statistical analyses, and wrote the manuscript. XD participated in the conception of the study, its design and coordination, and helped draft the manuscript. MS carried out the western blotting. LG assisted with cell proliferation assays and western blotting. BM assisted with cell proliferation assays and western blotting. DB helped draft and prepare the manuscript. MT and RB participated in the design and coordination of the study. TA participated in the conception and design of the study, participated in the statistical analysis, participated in the coordination of the study and edited the manuscript.
